# Powdery mildew resistance of apple clonal rootstocks from the collection of the Michurinsk State Agrarian University

**DOI:** 10.18699/VJGB-23-69

**Published:** 2023-10

**Authors:** I.N. Shamshin, M.L. Dubrovsky, A.A. Trifonova, K.V. Boris, A.M. Kudryavtsev

**Affiliations:** Michurinsk State Agrarian University, Michurinsk, Russia Vavilov Institute of General Genetics Russian Academy of Sciences, Moscow, Russia; Michurinsk State Agrarian University, Michurinsk, Russia Vavilov Institute of General Genetics Russian Academy of Sciences, Moscow, Russia; Vavilov Institute of General Genetics Russian Academy of Sciences, Moscow, Russia; Vavilov Institute of General Genetics Russian Academy of Sciences, Moscow, Russia; Vavilov Institute of General Genetics Russian Academy of Sciences, Moscow, Russia

**Keywords:** clonal rootstocks, Podosphaera leucotricha Salm., molecular markers, resistance genes, клоновые подвои, Podosphaera leucotricha Salm., молекулярные маркеры, гены устойчивости

## Abstract

Apple clonal rootstocks are the basis of modern intensive horticulture, providing a rapid increase in yield and convenience of fruit trees cultivation. Production of clonal rootstocks under high humidity often causes powdery mildew infection caused by the pathogenic fungus Podosphaera leucotricha Salm., which significantly reduces the productivity of stoolbed. Growing powdery mildew resistant genotypes is the most appropriate way to combat this disease and allows reducing the use of fungicides. To accelerate the search for resistant forms, molecular markers associated with resistance genes have been developed. However, these markers have not been used to study clonal rootstocks. The aims of the work were the field assessment of powdery mildew resistance of apple clonal rootstocks from the collection of the Michurinsk State Agrarian University and the screening of the collection for Pl-1, Pl-2, Pl-w and Pl-d resistance genes. The results of a three-year field evaluation of powdery mildew resistance of 80 rootstocks allowed us to distinguish five main groups ranging from very low to highly resistant. A group of 57 accessions was classified as powdery mildew resistant. The search for resistance genes was performed using the AT20 SCAR (Pl-1 gene), OPU02 SCAR (Pl- 2 gene), EM DM01 (Pl-d gene), and EM M02 (Pl-w gene) markers. The Pl-d and Pl-1 genes identified in 33 (41.25 %) and 31 (38.75 %) accessions, respectively, were the most common in the collection. The Pl-w gene was detected only in two accessions. Identification of the Pl-2 gene with the OPU02 SCAR marker did not reveal a fragment of the expected size. Thirty accessions with different powdery mildew resistance scores had two genes, Pl-1 and Pl-d, and highly resistant forms G16 and 14-1 had a combination of the Pl-d and Pl-w genes. These accessions can be used as donors of powdery mildew resistance for breeding new apple clonal rootstocks.

## Abstract (ru)

Клоновые подвои яблони являются основой современного интенсивного садоводства, они обеспечивают быстрое наращивание урожая и высокую технологичность возделывания плодовых деревьев. При производстве клоновых подвоев в условиях обильного увлажнения субстрата активно развивается возбудитель мучнистой росы – патогенный гриб Podosphaera leucotricha Salm., значительно снижающий продуктивность маточника. Использование устойчивых к мучнистой росе подвойных форм – наиболее целесообразный способ борьбы с этим заболеванием, позволяющий существенно сократить применение химических средств защиты растений. Для ускоренного поиска устойчивых форм разработаны молекулярные маркеры, ассоциированные с генами устойчивости. Однако для изучения клоновых подвоев эти маркеры ранее не применялись. Целью работы было изучение полевой устойчивости к мучнистой росе и выявление форм с генами устойчивости Pl- 1, Pl-2, Pl-w и Pl-d в крупнейшей отечественной коллекции клоновых подвоев яблони Мичуринского государственного аграрного университета. Результаты трехлетней полевой оценки устойчивости 80 отобранных для анализа подвойных форм позволили выделить пять основных групп образцов: от неустойчивых до высокоустойчивых. Наиболее многочисленной была группа устойчивых форм, включающая 57 образцов. Поиск генов устойчивости проводили с помощью маркеров AT20 SCAR (ген Pl-1), OPU02 SCAR (ген Pl-2), EM DM01 (ген Pl-d ) и EM М02 (ген Pl- w). Наибольшее распространение в изученной коллекции имеют гены Pl-d и Pl-1, обнаруженные у 33 (41.25 %) и 31 (38.75 %) образца соответственно. Ген Pl-w был выявлен только у двух форм, а при идентификации гена Pl-2 с маркером OPU02 SCAR не найдено фрагмента ожидаемого размера. При этом 30 образцов с различным уровнем полевой устойчивости имеют два гена, Pl-1 и Pl-d, а высокоустойчивые образцы G16 и 14-1 – комбинацию генов Pl-d и Pl-w. Эти образцы могут быть использованы в качестве доноров для создания новых клоновых подвоев яблони, несущих комплекс генов устойчивости к мучнистой росе.

## Introduction

Powdery mildew is one of the main fungal diseases of apple that cause significant economic damage to modern intensive horticulture. Powdery mildew is caused by the pathogenic fungus Podosphaera leucotricha Salm. The disease actively develops in humid warm weather in young orchards and nurseries. The fungus forms a white coating on the plant, which eventually turns brown. All above-ground parts of the plant (leaves, shoots, flowers and fruits) are affected by powdery mildew, leading to significant fruit yield losses (Kozlovskaya et al., 2018). During epiphytotic years, powdery mildew can affect up to 100 % of trees and lead to the loss of more than half of the yield. Up to twenty fungicide treatments a year are required to avoid severe powdery mildew infection, significantly increasing the chemical pressure on orchards (Holb et al., 2017; Höfer et al., 2021).

In our country, powdery mildew is widespread in the Southern Federal District (Yakuba, 2018), where intensive horticulture using dwarfing clonal rootstocks is developed. Due to the high regenerative and rooting ability of shoots and easy vegetative propagation, apple clonal rootstocks are one of the most important components in the production of planting material. Rootstocks provide tree anchorage, water and nutrient uptake and affect many other physiological processes. In modern industrial orchards, clonal rootstocks play a leading role in regulating tree vigor, precocity and also contribute to fruit quality and a rapid increase in yield. The use of dwarfing clonal rootstocks provides convenience of fruit tree cultivation, increases orchards productivity and is more economically efficient for fruit growers.

For the propagation of clonal rootstocks in stoolbeds, good rooting of shoots requires abundant moisture in the substrate, so systematic sprinkler irrigation is often used. Constant high humidity may lead to severe powdery mildew damage of the shoots of uterine bushes, causing a disruption in the normal functioning of the photosynthetic apparatus of leaves and a significant decrease in the productivity of the stoolbed. In this regard, many rootstock breeding programs are aimed at identifying new genotypes resistant to powdery mildew.

The search for sources of resistance to powdery mildew has been carried out since the second half of the XX century. To date, a number of resistance gene sources have been identified: the Pl-1 gene was first discovered in wild species Malus × robusta, the Pl-2 gene was identified in M. zumi (Knight, Alston, 1968), Pl-w in the cultivar White Angel (Gallott et al., 1985; Simon, Weeden, 1991), Pl-d in the hybrid D12 (Visser, Verhaegh, 1976) and Pl-m in the hybrid MIS (mildew immune selection) (Dayton, 1977).

To search for powdery mildew resistance gene sources, a number of markers have been developed and tested. Thus, the SCAR marker of the Pl-1 gene was created and successfully tested based on the RAPD marker OPAT20, and the SCAR marker of the Pl-2 gene, based on the RAPD marker OPU02 (Markussen et al., 1995; Gardiner et al., 2003). In addition, single nucleotide polymorphisms (SNPs) associated with Pl-2 powdery mildew resistance were identified and validated (Jänsch et al., 2015; Chagné et al., 2019). Based on the AFLP data, SCAR markers EM M01, EM M02 and EM DM01 of the Pl-w and Pl-d genes were created (Evans, James, 2003; James et al., 2004). Microsatellite markers CH03C02 and CH01D03 were also used to identify the Pl-d gene (James et al., 2004).

These markers have been successfully used to screen apple cultivar collections in Germany (Höfer et al., 2021) and the Czech Republic (Patzak et al., 2011), as well as to study wild Malus orientalis populations of Iran (Amirchakhmaghi et al., 2018). In our country, studies of the powdery mildew resistance genes distribution in commercial and local apple cultivars, as well as in wild species of the genus Malus have been carried out (Suprun et al., 2015; Lyzhin, Saveleva, 2020, 2021). However, apple rootstocks have barely been studied using powdery mildew resistance genes markers.

The Michurinsk State Agrarian University (Michurinsk SAU) is the founder of clonal rootstock breeding in Russia and one of the leading institutions in the world in this area. A unique collection of rootstocks, which is the largest in the country, is maintained here. Rootstocks bred at the Michurinsk SAU are grown in orchards in Russia, Europe, USA and other countries. However, there were no studies of their resistance to powdery mildew, including those using molecular markers. Such studies will help to identify sources and donors of resistance in the collection to create new rootstocks combining several powdery mildew resistance genes.

The aim of the work was to study field resistance of the Michurinsk SAU apple clonal rootstocks collection to powdery mildew and to identify accessions with the Pl-1, Pl-2, Pl-w and Pl-d resistance genes.

## Materials and methods

Eighty apple clonal rootstocks were studied, including 74 rootstocks of the Michurinsk SAU, three rootstocks from other Russian breeding centers (B7-35, K-1 and Ural-5) and three foreign rootstocks (M9 T337, G16 and Babarabskaya yablonya). Malus species from the collection of the N.I. Vavilov All-Russian Institute of Plant Genetic Resources (VIR), Belarusian cultivar Diyament and cultivar Evereste from the collection of the Michurinsk SAU were used as references for powdery mildew resistance genes Pl-1, Pl-2, Pl-d and Pl-w.

Total genomic DNA was extracted from fresh young leaves using the Quick-DNA Plant/Seed Miniprep Kit (Zymo Research, USA) following the manufacturers’ protocol.

Powdery mildew resistance genes were analyzed using the following DNA markers: codominant AT20 SCAR marker (Pl-1 gene) (Markussen et al., 1995), dominant OPU02 SCAR markers (Pl-2 gene) (Gardiner et al., 2003), EM DM01 (Pl-d gene) (James et al., 2004) and EM M02 (Pl-w gene) (Evans, James, 2003). The primers sequences and annealing temperatures are presented in Table 1.

**Table 1.**
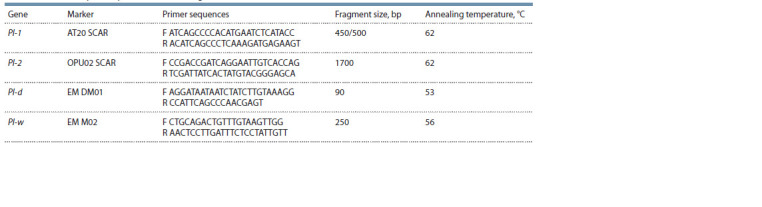
Markers for powdery mildew resistance genes identification

PCR reactions were performed in a SimpliAmp (Applied Biosystems, USA) thermal cycler in a final volume of 15 μl containing 20 ng of genomic DNA, 1.5 mM dNTPs, 2.5 mM MgSO4, 10 pM of each primer (Syntol, Russia), 1 u Taq polymerase and 1x standard PCR buffer (Thermo Fisher Scientific, USA). To determine fragment sizes, molecular markers GeneRuler 100 bp (Thermo Fisher Scientific), 50 bp DNA marker (Dialat Ltd, Russia) and Start 250 (Diaem, Russia) were used. After amplification, PCR products were separated in 2 % agarose gels, stained with ethidium bromide and analyzed on a UV-light box.

Powdery mildew resistance evaluation was carried out in the field for three years (2020–2022) in a nursery with sprinkler irrigation to maintain high substrate moisture. The evaluation was carried out according to the method described in the “Program and Methodology of Variety Studies for Fruit, Berry and Nut Crops” (Sedov, Ogoltsova, 1999), with minor modifications. The resistance rate was evaluated by the presence of powdery mildew lesions on the leaf blade. Ten shoots of each rootstock were analyzed. The assessment of the resistance rate was carried out on a five-point scale, where: 0 points – highly resistant genotype (HR, no lesions were detected), 0.1–1 points – resistant genotype (R, lesions up to 1 % of the leaf blade), 1.1–2 points – moderately resistant (M, lesions up to 10 % of the leaf blade), 2.1–3 points – low-resistant (L, lesions up to 40 % of the leaf blade), 3.1–5 points – very low-resistant (VL, lesions from 40 up to 100 % of the leaf blade). For each analyzed genotype, the average score for three years was calculated (Table 2).

**Table 2.**
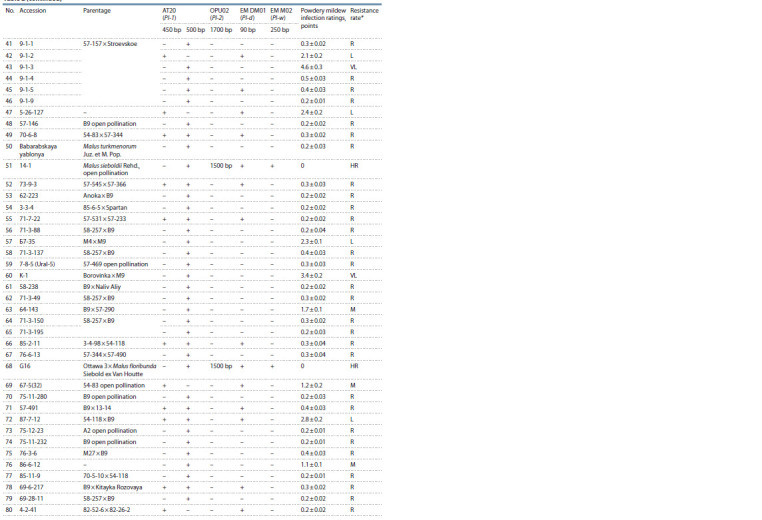
Analyzed apple rootstocks, data on the diversity of the Pl-1, Pl-2, Pl-d and Pl-w resistance genes and the results of field powdery mildew resistance assessment

**Table 2cont.**
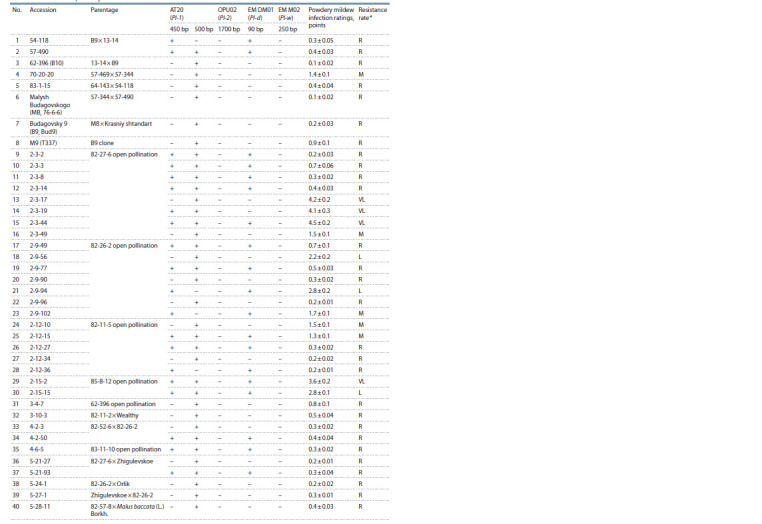
Table 2cont

**Table 2end.**
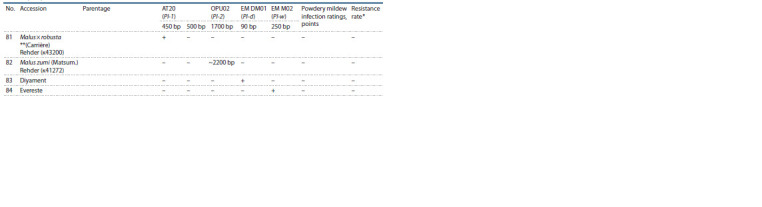
Table 2end * HR – highly resistant; R – resistant; M – moderately resistant; L – low-resistant; VL – very low-resistant. ** References for: Pl-1 gene – M. robusta (k43200); Pl-2 gene – M. zumi (k41272); Pl-d gene – Diyament; Pl-w gene – Evereste.

## Results

As a result of this work, 80 apple rootstocks were analyzed using markers of four powdery mildew resistance genes, Pl-1, Pl-2, Pl-w and Pl-d (see Table 2).

The Pl-1 gene was mapped in the resistance gene cluster of apple LG XII (Dunemann et al., 2007). To identify this gene, the SCAR marker AT20 was used, which allows detecting two fragments – 450 and 500 bp. The presence of a 450 bp fragment is associated with Pl-1 resistance (Markussen et al., 1995). The accession of wild species M. × robusta, from which this gene was initially introgressed, was used as reference.

**Fig. 1.**
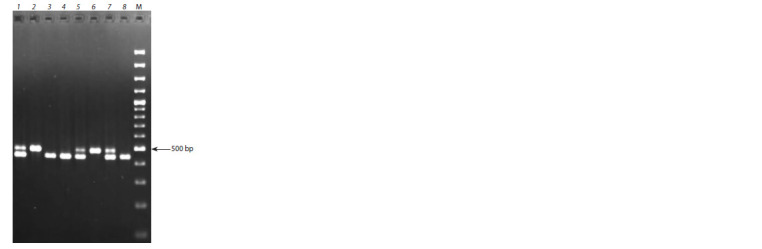
The results of the Pl-1 gene identification with the AT20 SCAR marker in apple clonal rootstocks. 1 – 57-490; 2 – B9; 3 – 54-118; 4 – 2-9-94; 5 – 2-12- 27; 6 – 5-27-1; 7 – 2-3-19; 8 – M. × robusta k43200. М – marker GeneRuler 100 bp.

Using the AT20 SCAR marker, a 450 bp fragment was detected in eight studied rootstocks: 54-118, 2-9-94, 2-9-102, 2-12-36, 4-2-41, 9-1-2, 5-26-127 and 67-5(32). At the same time, this fragment together with a 500 bp fragment was detected in 23 accessions, and in the remaining 49 accessions, only a 500 bp fragment was amplified (see Table 2). An example of Pl-1 gene identification is shown in Fig. 1.

The Pl-2 gene was mapped on apple LG XI. The OPU02 SCAR marker was used for Pl-2 identification (Gardiner et al., 2003). The presence of a dominant Pl-2 allele is detected by amplification of a 1700 bp PCR fragment (Gardiner et al., 2003). The accession of wild species M. zumi, from which this gene was initially derived, was used as reference.

**Fig. 2.**
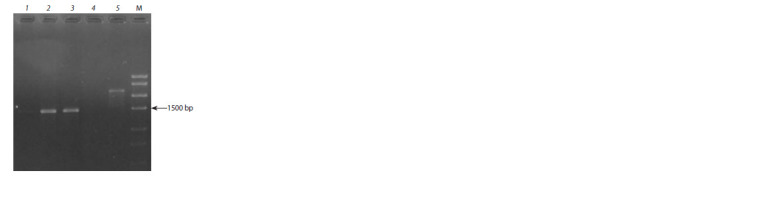
The results of the Pl-2 gene identification with the OPU02 SCAR marker in apple clonal rootstocks. 1 – 54-118; 2 – 14-1; 3 – G16; 4 – 9-1-2; 5 – M. zumi k41272. М – marker Start 250.

In the studied collection, an expected 1700 bp fragment was not revealed. However, in accessions 14-1 and G16, a 1500 bp fragment was amplified, while a ~2200 bp fragment was detected in the reference M. zumi accession (Fig. 2).

**Fig. 3.**
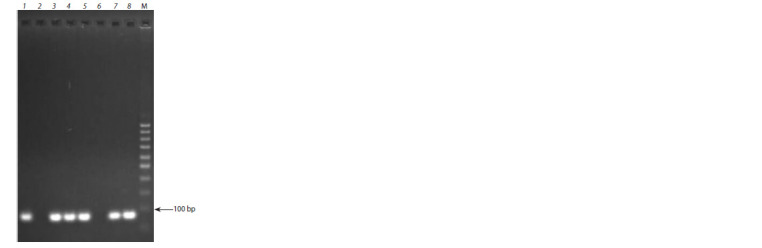
The results of the Pl-d gene identification with the EM DM01 marker in apple clonal rootstocks. 1 – 54-118; 2 – 62-396; 3 – 2-3-8; 4 – 2-3-14; 5 – 2-12-15; 6 – 2-12-10; 7 – 57-490; 8 – Diyament. М – 50 bp DNA marker.

The dominant SCAR marker EM DM01 was used to identify the Pl-d gene mapped on apple LG XII (James et al., 2004). The expected size of the PCR product of this marker is 90 bp. Apple cultivar Diyament, for which the presence of this gene was previously detected, was used as reference (Kozlovskaya et al., 2018). In the studied collection, the Pl-d gene was identified in 33 out of 80 analyzed accessions (see Table 2). An example of Pl-d gene identification is shown in Fig. 3.

**Fig. 4.**
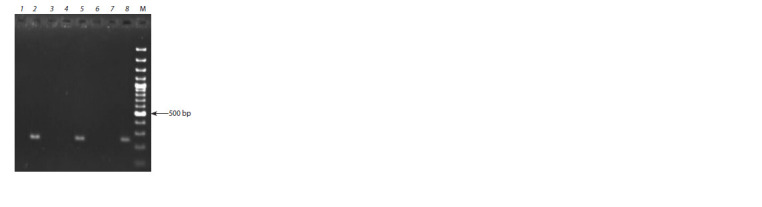
The results of the Pl-w gene identification with the EM M02 marker in apple clonal rootstocks. 1 – 2-3-17; 2 – 14-1; 3 – 7-8-5; 4 – 9-1-2; 5 – G16; 6 – 5-27-1; 7 – 2-3-8; 8 – Evereste. M – marker GeneRuler 100 bp.

The Pl-w gene was mapped on apple LG VIII. SCAR marker EM M02 was used for Pl-w identification (Evans, James, 2003). This marker amplifies a 250 bp fragment indicating the presence of the Pl-w gene. The Evereste apple, in which the Pl-w gene was previously identified, was used as reference (Patzak et al., 2011). In the studied collection of apple clonal rootstocks, the Pl-w gene was detected only in two accessions – G16 and 14-1 (see Table 2, Fig. 4)

**Fig. 5.**
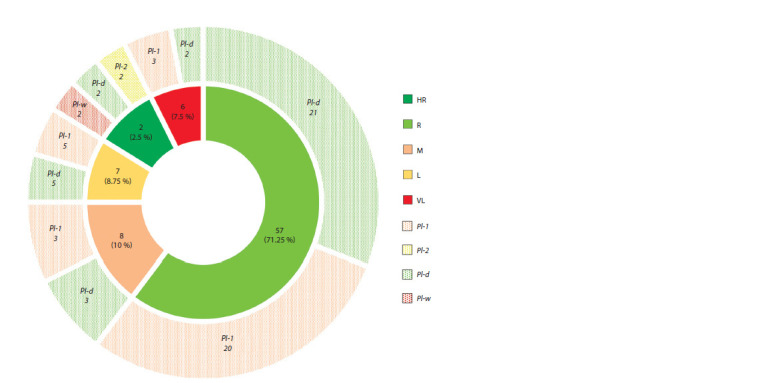
Proportion of accessions with different powdery mildew resistance in the studied collection according to field resistance evaluation data. Groups of accessions according to the field resistance rate: HR – highly resistant; R – resistant; M – moderately resistant; L – lowresistant; VL – very low-resistant (inner circle). The number of accessions with the Pl-1, Pl-2, Pl-w and Pl-d genes in each group (outer circle).

As a result of field evaluation of apple clonal rootstocks powdery mildew resistance, all studied accessions were divided into five main groups according to the resistance rate, from high to very low (Fig. 5).

It was shown that 57 accessions are resistant (0.1–1 points) to powdery mildew, which was 71.25 % of the total sample (see Fig. 5). In this group, the lowest average score of powdery mildew resistance (0.1) was noted in rootstocks 62-396 and 76-6-6, and the maximum (0.9), in rootstock M9 T337 (see Table 2). The group of moderately resistant forms (1.1–2 points) included eight accessions (10 %). The average resistance score for this group varied from 1.1 for 86-6-12 to 1.7 for rootstocks 2-9-102 and 64-143. The proportion of lowresistant (2.1–3 points) and very low-resistant (3.1–5 points) rootstocks was 8.75 %, (from 2.1 for rootstock 9-1-2 to 2.8 for rootstocks 2-9-94, 87-7-12 and 2-15-15) and 7.5 % (from 3.4 for K-1 to 4.6 for 9-1-3), respectively. The group of highly resistant accessions (0 points) was the smallest and included hybrid form 14-1 and rootstock G16 that had not been affected by powdery mildew (see Table 2, Fig. 5).

The distribution of the studied powdery mildew resistance genes in accessions with different field resistance was analyzed (see Fig. 5). The Pl-d and Pl-1 genes were the most widespread in the collection. Thus, the Pl-d gene was present in all groups, and the Pl-1 gene was not detected only in highly resistant accessions. While the Pl-w gene, on the contrary, was detected only in two accessions 14-1 and G16, which are highly resistant. In addition, specific 1500 bp PCR products were detected only in these accessions with the Pl-2 gene marker (see Fig. 2).

## Discussion

The analysis of 80 apple clonal rootstocks using markers of powdery mildew resistance genes Pl-1, Pl-2, Pl-d and Pl-w allowed assessing the variability of these genes in the Michurinsk SAU collection for the first time.

The most common in the studied collection were the Pl-d and Pl-1 genes identified in 33 (41.25 %) and 31 (38.75 %) accessions, respectively (see Table 2). The Pl-w gene was detected only in G16 and 14-1 accessions (see Table 2, Fig. 4). The distribution of the studied powdery mildew resistance genes in the collection may be related to the rootstocks pedigrees, including both wild Malus species and commercial apple cultivars and landraces (see Table 2).

Wild apple species are the most promising sources of disease and pest resistance genes for breeding apple cultivars and rootstocks (Pereira-Lorenzo et al., 2018; Solomatin, 2018). However, when breeding clonal rootstocks, during the primary selection of hybrid seedlings, genotypes with good vegetative propagation ability, high rooting ability and increased winter hardiness of the root system are distinguished. The study of resistance to phytopathogens is usually carried out later among the selected rootstocks.

It should be noted that there were practically no studies of apple rootstocks collections using powdery mildew resistance gene markers, and the researchers have been mainly focused on studying apple cultivars and wild Malus species. For example, the study using the EM DM01 marker showed a wide distribution of the Pl-d gene among old and modern apple cultivars from the collection of the Institute for Fruit Growing, Belarus (Urbanovich et al., 2010). In the study of 145 old and local apple cultivars from the Czech collection, the Pl-d gene was detected only in four accessions (Patzak et al., 2011). The study of apple cultivars from the Dresden- Pillnitz gene bank using the microsatellite marker of the Pl-d gene also showed its low distribution (Höfer et al., 2021).

The Pl-d gene was also found in wild apple species. In the study of 67 Malus species forms, the Pl-d gene was identified in seven accessions, and for M. sieversii and M. orientalis, intraspecific polymorphism was revealed (Lyzhin, Saveleva, 2021). In the study of M. orientalis populations growing in Iran, the Pl-d gene diversity was also noted (Amirchakhmaghi et al., 2018). Transcaucasia is thought to be one of the centers of origin of low-vigorous clonal rootstocks, where they originated from wild species (Budagovskiy, 1976; Solomatin, 2018). The most common apple species growing in this region is M. orientalis. Thus, the distribution of the Pl-d gene in the collection may be associated with the origin of rootstocks from wild species, including M. orientalis. Another possible factor of the distribution of this gene in the analyzed collection is the use of apple cultivars in breeding rootstocks.

The Pl-1 gene was the second most common gene in the collection. According to previous studies, this gene is not widely distributed, both among old and commercial apple cultivars (Urbanovich et al., 2010; Patzak et al., 2011; Suprun et al., 2015; Kozlovskaya et al., 2018; Lyzhin, Saveleva, 2020; Höfer et al., 2021). However, the Pl-1 gene was found in Malus species. In a collection of 67 wild Malus forms, the Pl-1 gene was detected in 37.3 % of accessions, including M. × robusta, the species this gene was initially derived from. At the same time, intraspecific polymorphism for this gene was shown for some species (Lyzhin, Savelyeva, 2021). The presence of the Pl-1 gene in wild species was also noted in the study by O. Urbanovich et al. (2010). Apparently, the distribution of the Pl-1 gene in the collection of apple clonal rootstocks is associated with the presence of wild Malus species in their pedigrees.

The Pl-w gene, identified in two rootstocks using the EM M02 marker, is less distributed in the studied collection. The Pl-w gene provides a higher level of resistance than the Pl-1 and Pl-2 genes (Simon, Weeden, 1991; Evans et al., 2003). This gene is quite common among wild Malus species, but is practically not found in apple cultivars (Patzak et al., 2011; Kozlovskaya et al., 2018; Lyzhin, Saveleva, 2021).

Probably, it is the presence of the Pl-w gene that ensures high powdery mildew resistance in G16 and 14-1 accessions, which was revealed during the field resistance assessment (see Table 2). Apparently, this is due to their origin from wild species. The American rootstock G16 is derived from M. flo-ribunda, and the hybrid form 14-1 of the Michurinsk SAU is derived from M. sieboldii. Previously, using the EM M02 marker, the Pl-w gene was detected in M. floribunda and M. sieboldii (Lyzhin, Saveleva, 2021).

The Pl-2 gene marker OPU02 SCAR revealed ~1500 bp fragments in two accessions (G16 and 14-1) from the collection (see Fig. 2), while the presence of a dominant Pl-2 allele was detected by amplification of a 1700 bp fragment (Gardiner et al., 2003). Previously, using the OPU02 SCAR marker, a 1500 bp fragment was detected in the cultivar Favorit of the Crimean Experimental Horticultural Station (Suprun et al., 2015). The presence of unknown alleles of this gene or a new resistance gene was suggested, as the cultivar is powdery mildew resistant. This may also refer to accessions G16 and 14-1, since both are completely resistant to powdery mildew according to the results of the field evaluation, although these accessions also have the Pl-d and Pl-w genes (see Table 2).

The Pl-2 gene was first identified in M. zumi (Knight, Alston, 1968). However, in the M. zumi accession from the VIR collection used as reference for this gene, a fragment of ~2200 bp was obtained, instead of the expected 1700 bp fragment (see Fig. 2). This may be due to the intraspecific polymorphism, previously noted for other powdery mildew resistance genes in wild Malus species (Lyzhin, Saveleva, 2021). Alternatively, the presence of a ~2200 bp fragment may be related to the existence of another powdery mildew resistance gene Pl-MIS in the M. zumi accession (Gardiner et al., 2003).

The results obtained using the OPU02 SCAR marker do not allow to identify the Pl-2 gene by the presence of the 1700 bp fragment, which may be due to a significant distance (8.6 cM) from the marker to the gene (Gardiner et al., 2003). Therefore, in order to assess the presence of the Pl-2 gene in the collection, other markers can be used, e. g. SNPs (Jänsch et al., 2015; Chagné et al., 2019).

It is known that in breeding for resistance, a common approach is to combine several resistance genes in one genotype, the so-called “pyramiding”. The results allowed to identify a number of rootstocks that are promising for breeding. In the studied collection, 30 accessions have two resistance genes, Pl-1 and Pl-d, and accessions G16 and 14-1, highly resistant to powdery mildew, have genes Pl-d, Pl-w, as well as a specific fragment detected using the Pl-2 gene marker (see Table 2).

However, not all accessions with powdery mildew resistance genes showed high field resistance. For example, all eight accessions that had the dominant allele of the Pl-1 gene also had the Pl-d gene, however, the level of their field resistance varied from 0.2 points for rootstock 4-2-41 to 2.8 points for rootstock 2-9-94 (see Table 2). At the same time, accessions G16 and 14-1 that had the Pl-d and Pl-w genes, as well as a specific fragment obtained with the Pl-2 gene marker, were highly resistant and had no symptoms of infection (0 points).

The discrepancy between field resistance level and the presence of resistance genes can have several causes. For some of the markers, there are conflicting data on the association with the trait. A number of studies do not confirm the correlation of Pl-1 AT20 SCAR and Pl-d EM DM01 markers with resistance (Dunemann et al., 2004; Kellerhals et al., 2008; Urbanovich et al., 2010).

In addition, data on the presence and distribution of different races of P. leucotricha and the results of artificial inoculation tests with known pathogen strains are needed for more accurate assessment of the powdery mildew resistance level. Besides, the resistance determined by known Pl genes can be overcome, as, for example, Pl-1 gene resistance (Lesemann, Dunemann, 2006). Furthermore, despite a significant amount of data on the genome sequences of the cultivated apple and other Malus species, it is likely that not all powdery mildew resistance genes and alleles of known resistance genes have been identified at the moment.

## Conclusion

Field evaluation of powdery mildew resistance of 80 accessions from the Michurinsk SAU apple clonal rootstocks collection was assessed for the first time and accessions with powdery mildew resistance genes were identified. The Pl-d and Pl-1 genes were the most common in the collection. The Pl-w gene was found only in two accessions, while the Pl-2 gene was not detected in the collection. At the same time 32 accessions had a combination of two Pl genes. The presence of powdery mildew resistance genes in rootstocks appears to be related to their pedigrees and origin from wild Malus species. Comparison of the field resistance assessment results with the molecular analysis data showed that the presence of resistance genes does not always correspond to the level of resistance. Thus, the Pl-d and Pl-1 genes were identified in both resistant and low-resistant accessions, while the Pl-w gene was detected only in highly resistant accessions 14-1 and G16. The obtained data can be used for breeding new apple rootstocks with a combination of powdery mildew resistance genes.

## Conflict of interest

The authors declare no conflict of interest.
